# Supplementary figures and images for: Cost-efficient vaccination protocols for network epidemiology
Source: PLoS Comput Biol. 2017 Sep 11;13(9):e1005696. doi: 10.1371/journal.pcbi.1005696 (PMC5608431; doi:10.1371/journal.pcbi.1005696)

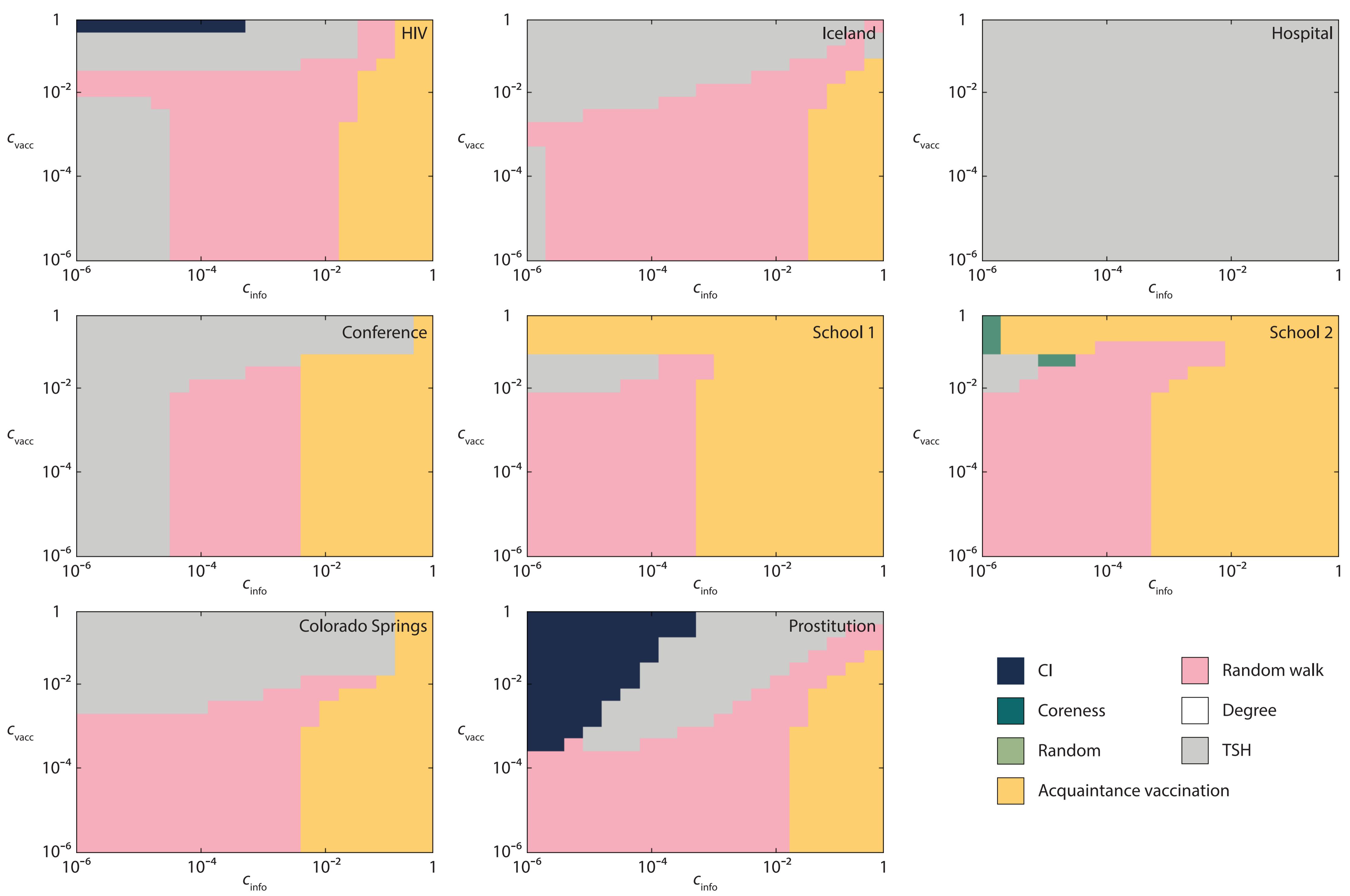

Supplement: S1 Fig — The figure corresponding to Fig 4, but for β = 1/2. (PDF) [file pcbi.1005696.s001.pdf]

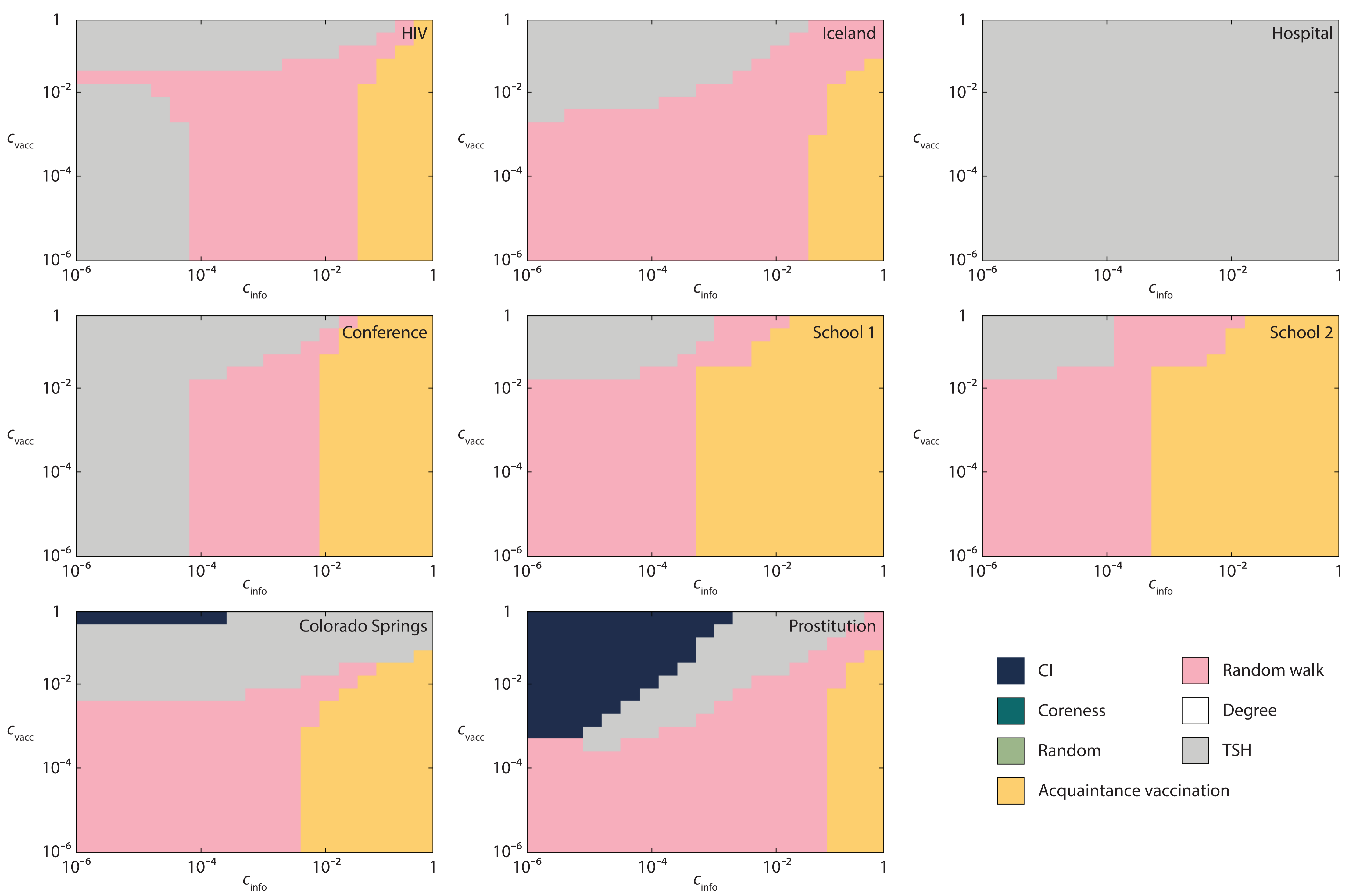

Supplement: S2 Fig — The figure corresponding to Fig 4, but for β = 1. (PDF) [file pcbi.1005696.s002.pdf]

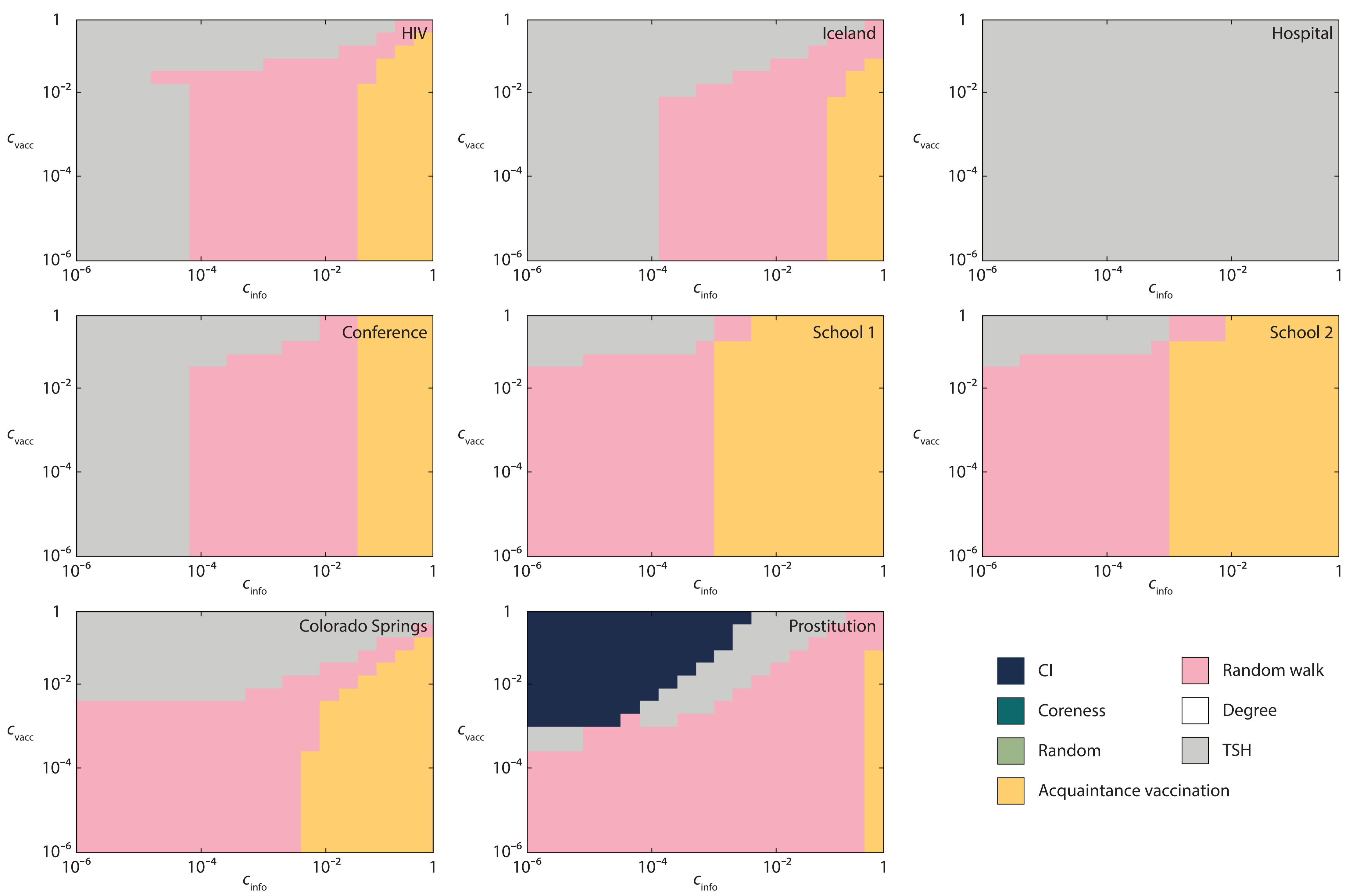

Supplement: S3 Fig — The figure corresponding to Fig 4, but for β = 4. (PDF) [file pcbi.1005696.s003.pdf]

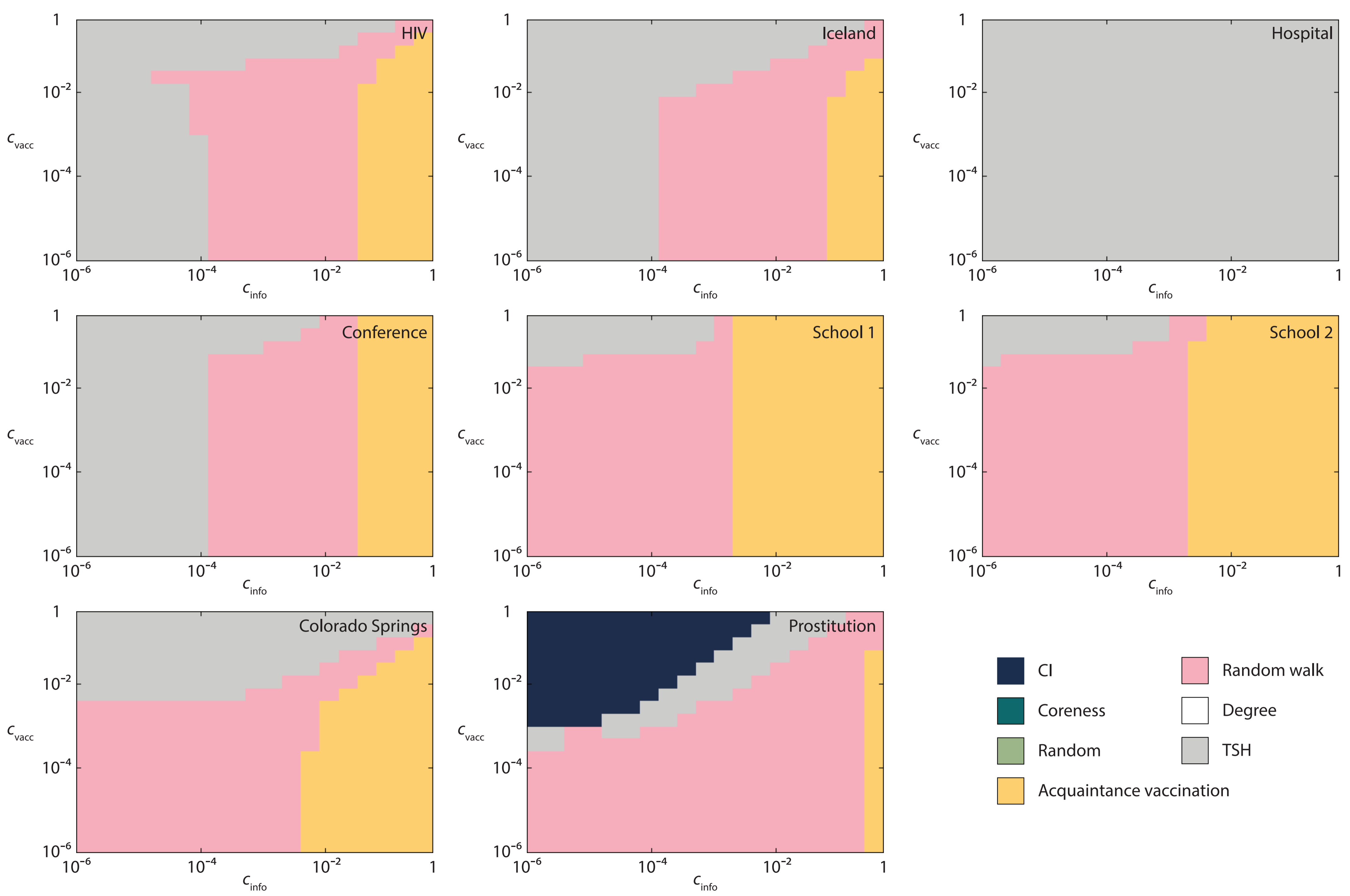

Supplement: S4 Fig — The figure corresponding to Fig 4, but for β = 8. (PDF) [file pcbi.1005696.s004.pdf]

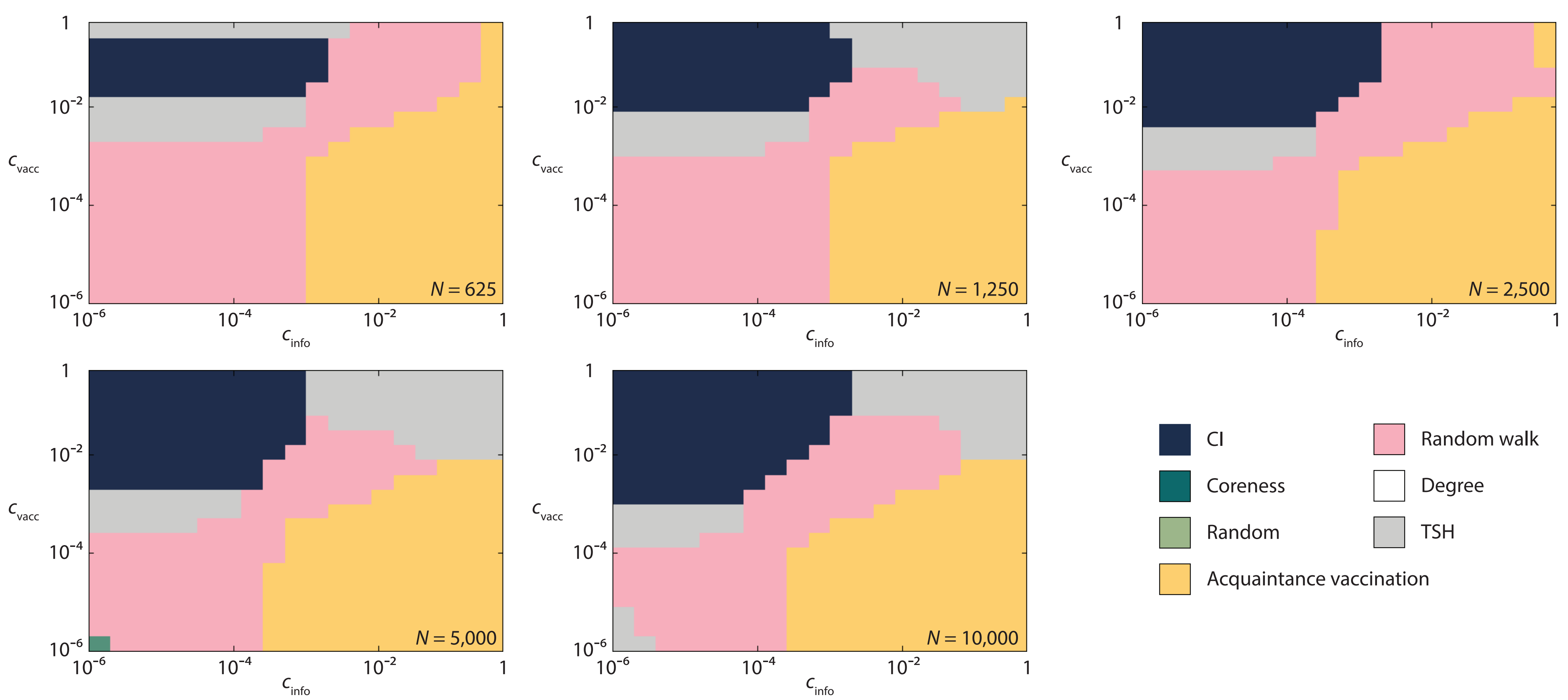

Supplement: S5 Fig — The figure corresponding to Fig 5, but for β = 1/2. (PDF) [file pcbi.1005696.s005.pdf]

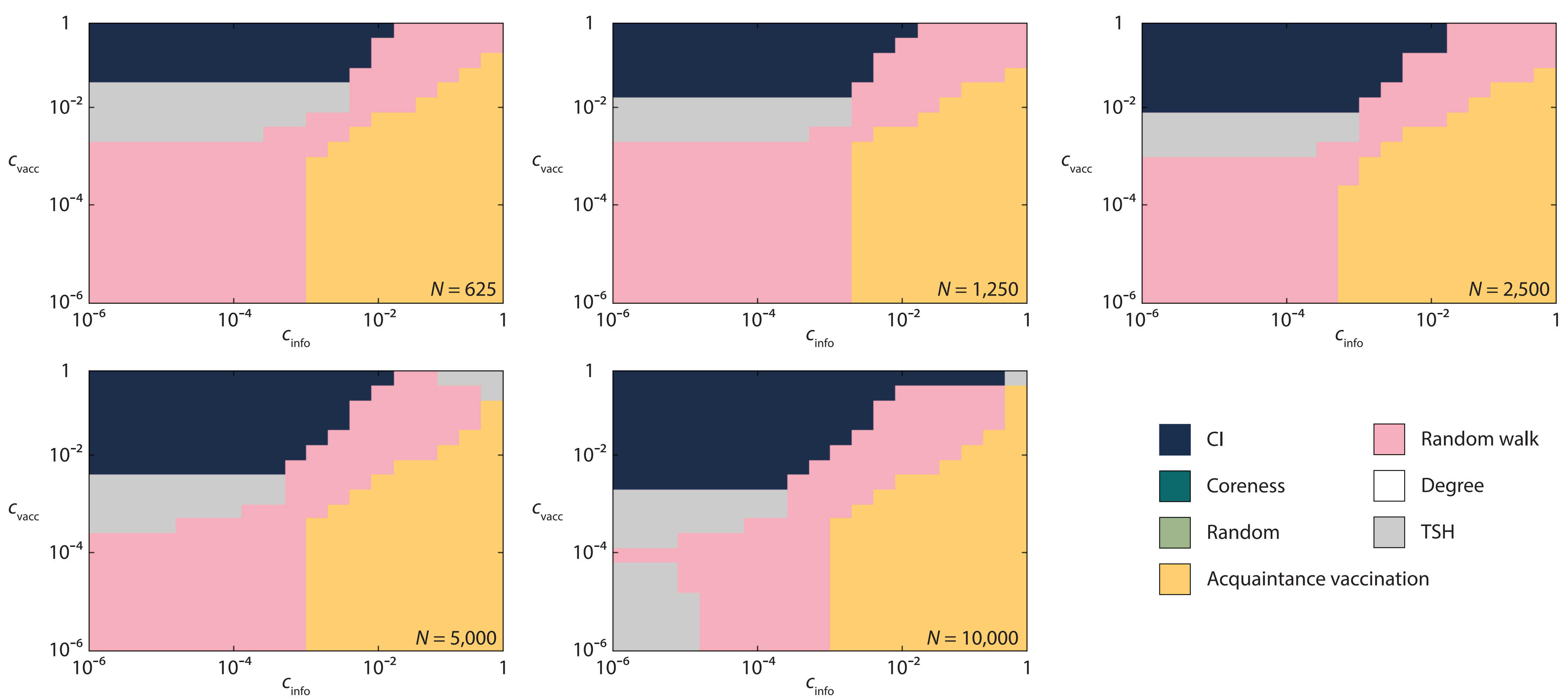

Supplement: S6 Fig — The figure corresponding to Fig 5, but for β = 1. (PDF) [file pcbi.1005696.s006.pdf]

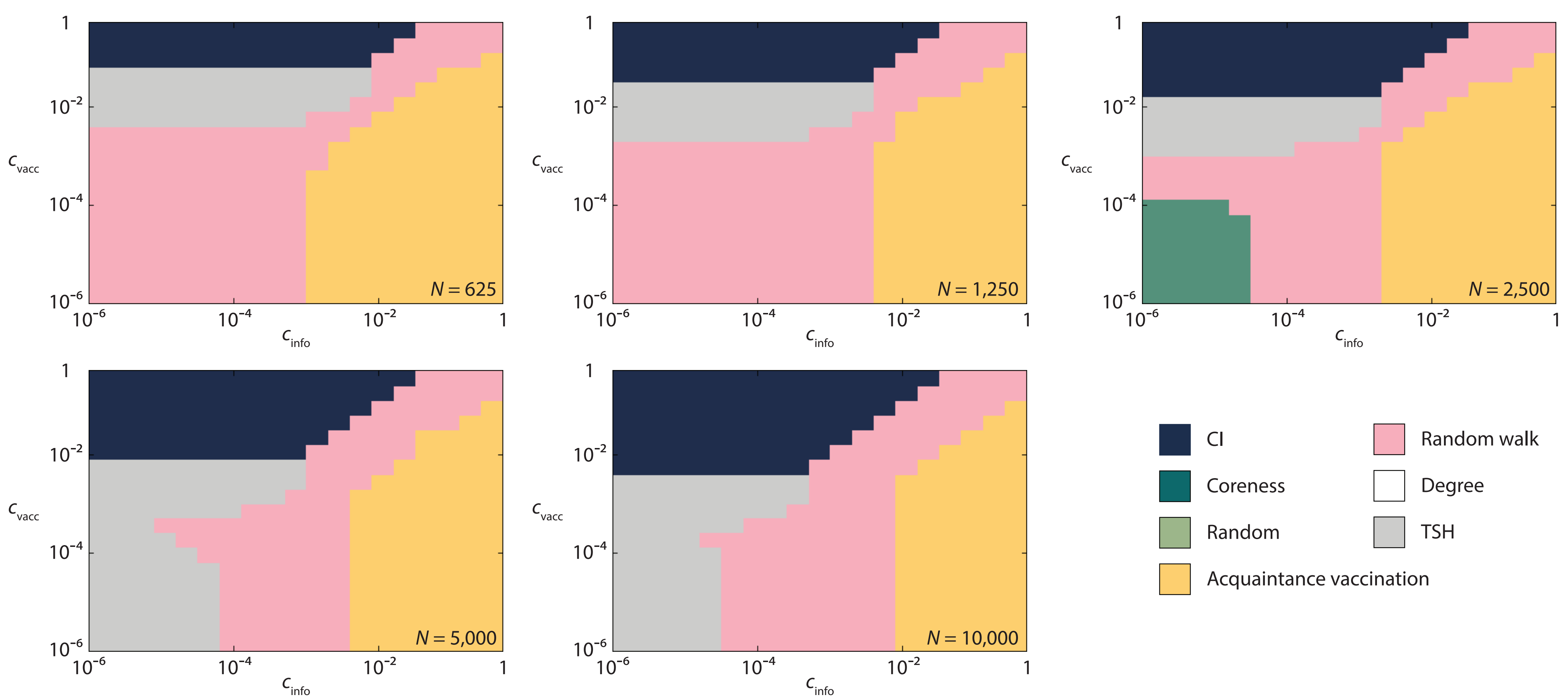

Supplement: S7 Fig — The figure corresponding to Fig 5, but for β = 4. (PDF) [file pcbi.1005696.s007.pdf]

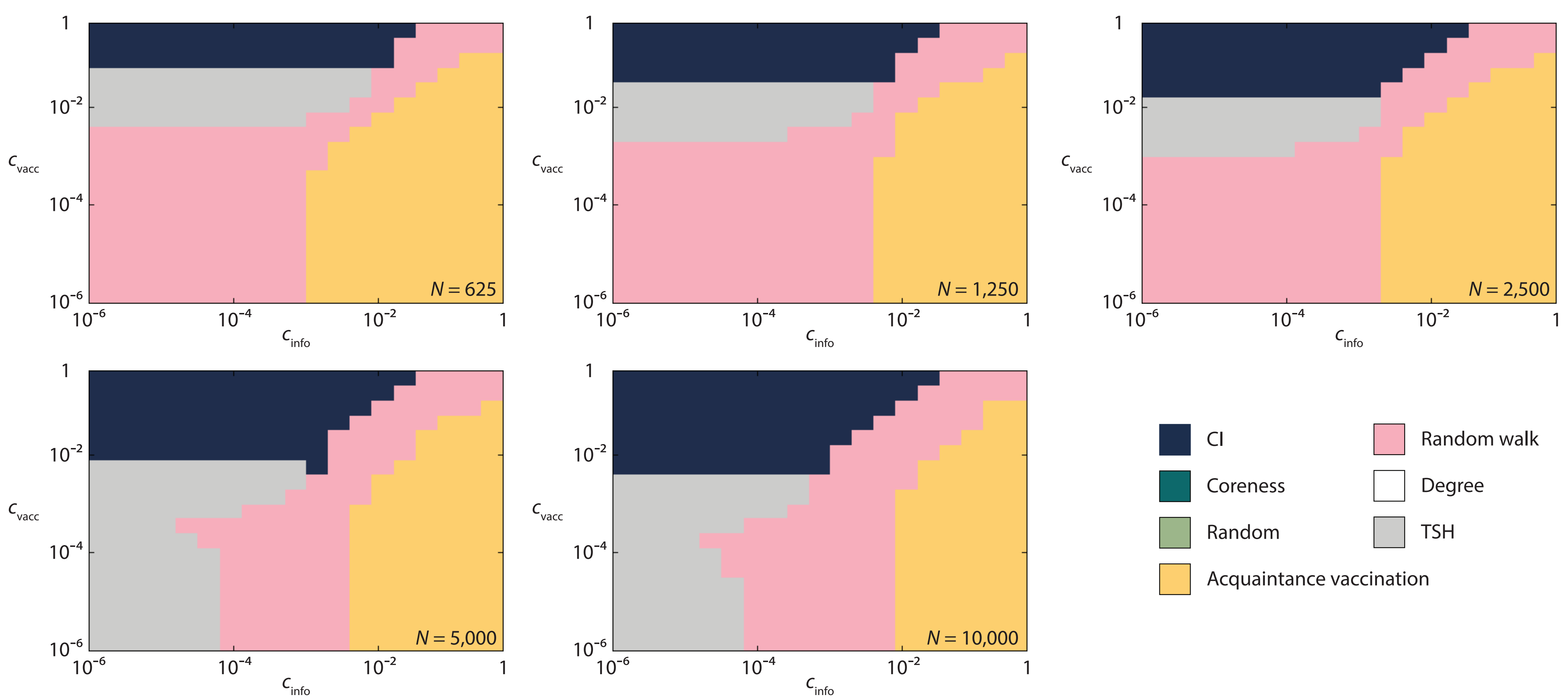

Supplement: S8 Fig — The figure corresponding to Fig 5, but for β = 8. (PDF) [file pcbi.1005696.s008.pdf]
